# Neutrophil Extracellular Traps and Pancreatic Cancer Development: A Vicious Cycle

**DOI:** 10.3390/cancers14143339

**Published:** 2022-07-08

**Authors:** Michail Mitsis, Panagiota Drosou, Vasileios Tatsis, Georgios S. Markopoulos

**Affiliations:** 1 Department of Surgery, School of Health Sciences, Faculty of Medicine, University Hospital of Ioannina, University of Ioannina, 455 00 Ioannina, Greece; mmitsis@uoi.gr (M.M.); panagiotadrosou@gmail.com (P.D.); tatsis.vasileios@hotmail.com (V.T.); 2 Cancer Biobank Center, University of Ioannina, 455 00 Ioannina, Greece; 3 Haematology Laboratory-Unit of Molecular Biology, University Hospital of Ioannina, 455 00 Ioannina, Greece

**Keywords:** NETs, NETosis, hypercoagulability, pancreatic adenocarcinoma, inflammation, cancer

## Abstract

**Simple Summary:**

Cancer-associated thrombosis is a significant complication in cancer patients with various clinical consequences. Neutrophil extracellular traps (NETs), are associated with cancer development and hypercoagulability in various cancers, including pancreatic cancer. On the other hand, pancreatic cancer development is associated with increased NETs formation. This feedback mechanism suggests the existence of a vicious cycle between NETs and the tumor microenvironment in pancreatic cancer, that supports tumor development and may lead to cancer-associated thrombosis.

**Abstract:**

Neutrophil extracellular traps (NETs) are a neutrophil-generated extracellular network of chromatin and chromatin-bound molecules with antimicrobial potency. Recent data suggest that NETs are associated with cancer progression and cancer-associated hypercoagulability. Pancreatic adenocarcinoma (PDAC) is a lethal type of cancer in which hypercoagulability and cancer-related thrombosis are among the main complications. In the current report, we summarize the available data on the interplay between NET formation and PDAC development. We conclude that NETs support a dual role during PDAC progression and metastasis. Their formation is on the one hand an important event that shapes the cancer microenvironment to support cancer cell proliferation, invasion and metastasis. On the other hand, NETs may lead to cancer-associated thrombosis. Both mechanisms seem to be dependent on distinct molecular mechanisms that link inflammation to cancer progression. Collectively, NET formation may contribute to the pathogenesis of PDAC, while during cancer development, the proinflammatory environment enables the induction of new NETs and thrombi, forming a vicious cycle. We suggest that targeting NET formation may be an effective mechanism to inhibit both PDAC development and the accompanying hypercoagulability.

## 1. Introduction

Cancer is a major health concern and among the leading causes of human mortality worldwide. It has been estimated that more than 19 million new cancer cases and about 10 million cancer-related deaths occurred during 2020. Future estimations remain dismal, with a projected rise of >70% in both incidence and mortality in the next 20 years, leading to cancer-related incidence and mortality to reach >28 and >16 million, respectively, in 2040 [1]. Among the types of cancer, pancreatic ductal adenocarcinoma (PDAC/ICD code C25) accounts roughly for 500,000 new cancer cases. PDAC is among the deadliest types of neoplasms, based on the high mortality rate of at least 90%, since 460,000 deaths have been estimated to have occurred in 2020. PDAC is commonly an aggressive type of cancer and the overall 5-year survival rate remains significantly low at only 6% [1]. Among the main complications of PDAC is the induction of cancer-associated thrombosis, which affects quality of life and disease progression [2]. In particular, it is a well-established concept that PDAC induces a hypercoagulable state that ultimately leads to thrombosis. There are several proposed mechanisms that connect the phenomena of PDAC and thrombosis, including the high expression of tissue factor (TF), the activation of specific subpopulations of white blood cells, the spreading of microvesicles from cancer cells as well as the release of neutrophil extracellular traps (NETs) [2].

NETs are extracellular structures that contain decondensed chromatin and chromatin-bound cytosolic and granule proteins, including several enzymes, formed by neutrophils [3]. It has been suggested that the main biological role of NETs is to entrap and neutralize microorganisms, avoiding their further propagation [4]. Conversely, a dysregulation in NET formation can contribute to several types of pathology, including immune-related and inflammatory disease, as well as cancer [4,5,6,7].

NETs have several possible roles in the gastrointestinal system, from the intensification of inflammation and the development of autoimmune response to the induction of cancer metastasis and cancer-associated thrombosis [8,9]. Importantly, the formation of NETs, the interactions between NETs and microorganisms as well as between NETs and neutrophil subtypes affect their final biological outcome [10,11]. In addition, there are several NET-associated active biological molecules, which include cell-free DNA and citrullinated histone proteins, which have the potential of being used as specific biomarkers of disease in the gastrointestinal system and/or as therapeutical targets [12].

Among others, two significant associations between NETs and cancer are: (a) the induction of hypercoagulability, leading to cancer-associated thrombosis and (b) the promotion of cancer progression through several mechanisms, such as the induction of invasion and metastasis and the maintenance of a proinflammatory state [13]. In this review paper, we thoroughly discuss the above concepts, giving an emphasis to PDAC development and therapy.

## 2. Neutrophils and Induction of NETs in Physiology and Pathology

NETs were first characterized in 2004 by Brinkmann and colleagues, who observed that acute bacterial infection leads to neutrophil activation and the release of extracellular structures resembling networks which contain chromatin fibers and proteases such as neutrophil elastase [3]. Subsequent studies revealed that, except bacteria, NET formation can also be the result of infection from fungi, viruses and parasites [4,14,15,16]. The antimicrobial role of NETs has been confirmed by studies that showed the ability of NETs to prevent microbial dissemination [17] as well as the interaction between NETs and platelet Toll-like receptor 4 (TLR4) during sepsis [18]. Later, it was shown that the formation of NETs represents the result of a specialized, regulated type of neutrophils’ cell death, different from both necrosis and apoptosis, a process which has been called NETosis [19]. NETosis leads to the externalization of the cellular content of neutrophils, including chromatin, and has the ability to act as a trap to microorganisms in a manner similar to a “spider-web” [19]. NETs have since been further characterized as an extracellular network of chromatin fibers that also embed proteases for the purpose of trapping and eliminating microbes. In addition to their antimicrobial roles, the elastase and myeloperoxidase of neutrophils also play regulatory roles in the formation of NETs [20,21].

Recent data suggest that the creation of these structures can occur either gradually, starting their assembly intracellularly [19], or in other cases, as is usually the case during sepsis or following acute infections, after rapid secretion and extracellular assembly following NETosis [22,23]. Regardless of the assembly mechanism, the structural characteristics of NETs remain the same, allowing the study of both types of networks through the adoption of similar methods [4].

In addition to the physiological roles in defense against microbial infections, uncontrolled NET formation plays important roles in inflammation, autoimmunity and cancer [5,6]. Recent data suggest that NETs may play regulatory roles that lead to the “awakening” of dormant cancer cells [24], the promotion of metastases, the evasion of immune response to cancer [9,25,26] and also in the development of cancer-associated thrombosis [27].

## 3. NETs and PDAC Progression

PDAC is a poor-prognosis cancer in which neutrophils are aberrantly recruited in its microenvironment, driving tumorigenesis [28]. An induced formation of NETs in the pancreatic tumor microenvironment has been associated with the development and the progression of PDAC [29], involving several molecules and mechanisms.

A proposed mechanism that supports the role of NETs in PDAC includes the overexpression of the tissue inhibitor metalloproteinases-1 (TIMP1). A study by Schopes et al. revealed that TIMP1 protein expression most prominently correlates with neutrophil activation in patient-derived tumor tissues. Following the activation of neutrophils, TIMP1 triggered the formation of NETs by interacting with its receptor CD63 and by subsequently inducing the MEK/ERK signaling pathway. The aforementioned study also proved that NET formation in a PDAC mouse model is related to TIMP1 overexpression, while the abolition of either TIMP1 expression or NET formation is associated with prolonged survival. Importantly, in patient-derived PDAC tumors, several lines of evidence supported the connection between NET formation and TIMP1 expression, including: the colocalization of NETs with areas of elevated TIMP1 expression; the correlation of plasma levels of TIMP1 with the NETs marker myeloperoxidase and the identification of prognostically distinct PDAC subgroups associated with CA19-9 marker expression [29]. This seminal study is of great importance, given also that the overexpression and secretion of TIMP1 is inextricably linked to the development of PDAC [30]. TIMP1 has as well been proposed as a potential serum marker for the detection of familial PDAC in its early stages [31], alone or in combination with long non-coding RNA LNC2 and the microRNA miR-196b [32].

Inflammation is associated with NET formation and cancer progression. Inflammatory response may lead to NET formation and vice versa [33]. Similarly, mediators of inflammation such as cytokines are part of regulatory networks that allow the interplay between inflammation and tumorigenesis [34]. Among the pro-inflammatory cytokines, interleukin-17 (IL-17) is associated with cancer formation and host defense against several types of infection [35]. Zhang et al. have shown that IL-17 is involved in pancreatic tumorigenesis by recruiting neutrophils and inducing NET formation, which subsequently excludes the penetrance of cytotoxic CD8+ T lymphocytes from the tumor microenvironment. The induced expression of IL-17 is also related to poorer prognosis and a higher potential for NETosis in patients with PDAC. The authors suggested that a combinatorial therapy that will target both IL-17 and a checkpoint blockade will have a potential efficacy for PDAC treatment [36].

Platelet-activating factor (PAF) stimulates a signaling cascade that has evolved both as a component of innate host defense and as an effector pathway in inflammation and thrombosis, in either physiological or pathological conditions [37]. Boone et al. found that following stimulation with PAF, neutrophils isolated from animal models of PDAC had a tendency to form NETs, together with elevated serum DNA and citrullinated histone H3 expression, which are markers of NET formation [38]. These results were also confirmed in peripheral blood serum from patients with PDAC. Significantly, the proposed mechanism of NET induction was found to be dependent on autophagy induction and the activation of the receptor for advanced glycation end products (RAGE) signaling. The inhibition of autophagy by chloroquine or the genetic ablation of RAGE resulted in reduced NET formation, which was monitored by decreased serum DNA and citrullinated histone H3 expression [38]. The importance of RAGE signaling in the formation of NETs is supported by its central role in tumor-related inflammation [39], in part through the induction of the transcription factors of the NF-κB family [40]. The interplay between autophagy and NETosis is an additional mechanism that may link inflammation to cancer progression [41]. As regards pancreatic cancer development, the role of autophagy is essential, since it is required for tumor growth [42]. Owing to this, it has been suggested that autophagy inhibition may be a potent therapy for PDAC [43]. We believe that this finding might in part be associated with the induction of NETosis.

The creation of NETs leads to the secretion of several neutrophil-derived proteins and chromatin into the extracellular space within the tumor microenvironment, a process that is mediated by peptidyl arginine deiminase 4 (PADI4) and the entrance of neutrophil elastase to the nucleus [44]. NETs promote PDAC proliferative potential and metastatic competence. Miller-Ocuin et al. have demonstrated that in a PDAC mouse model, Padi4 ablation resulted in decreased PDAC growth, diminished pancreatic stromal activation and a reduction in circulating extracellular DNA levels, an indication of reduced NET formation. In contrast, cell-free DNA from neutrophils resulted in the activation of pancreatic stellate cells and the formation of a tumor-assisting stroma that promotes cancer cell proliferation. This effect has been specifically inhibited following DNase treatment. To corroborate on this result, serum derived from PDAC patients has been correlated with the tumor stage. The authors also proved that the effect of NETs in pancreatic stellate cells is mediated by RAGE signaling. Collectively, NET formation has been associated with PDAC development in a PADI4- and RAGE-dependent manner [44]. This significant association highlights NET formation as a potentially attractive therapeutic strategy in patients with PDAC.

NETs, as part of tumor stroma, support cancer cell growth and development [45]. The tumor microenvironment consists a number of non-tumor cells, such as cancer-associated fibroblasts (CAFs) that may sustain tumorigenesis by acting to some extent as modulators of immune responses [46,47]. Recent research supports a connection between NETs and CAFs, in which CAFs may attract neutrophils and induce NET formation. The proposed mechanism includes the induction of amyloid-β by CAFs that results in NET formation in a reactive oxygen species (ROS)-dependent manner [48]. Amyloid-β is a peptide that is implicated in both neurodegenerative and inflammatory diseases [49]. Amyloid-β has been found upregulated in cancer patients, more prominently in PDAC [50]. Interestingly, in a murine PDAC model, tumor growth was dependent on CAF-derived NETs, as shown by inhibiting NETosis. CAFs were present in parallel to NET formation in human PDAC as well as in melanoma cancer, while induced expression of amyloid-β and β-Secretase has been correlated with poor prognosis [48]. Additional lines of evidence support the interplay between CAFs and NETs in PDAC development. A recent report has revealed that PDAC micrometastasis in the liver tissue is induced following NET formation, a process dependent on the activation of CAFs [51]. CAFs and NETs may also influence the immune modulation of the tumor microenvironment, a phenomenon that contributes to the pathogenesis of pancreatic cancer [52].

Given the importance of the tumor microenvironment in cancer development [53,54], it is a reasonable conclusion that the presence of tumor-infiltrating NETs may predict post-surgical survival in patients with PDAC [55], a common characteristic shared with tumor-infiltrating platelets [56]. A molecular mechanism that explains NET-associated epithelial to mesenchymal transition (EMT), migration and metastasis includes the activation of the EGFR/ERK signaling pathway [28]. This mechanism is dependent on the expression of the inflammatory cytokine IL-1β. This regulatory role of IL-1β is in accordance with experiments in a NETs-dependent breast cancer model, in which the inhibition of IL-1β leads to the attenuation of thrombosis [57].

A critical factor that can induce invasion and metastasis in PDAC is collagen deposition [58], which is also a factor related to poor prognosis [59]. Signaling pathways resulting in collagen deposition are associated with the activating receptor tyrosine kinase discoid domain receptor 1 (DDR1) [60]. DDR1 has the potential to induce neutrophil extracellular traps that promote PDAC metastasis [61]. Collagen-induced DDR1 activation in PDAC may lead to the production of the chemokine CXCL5, the recruitment of neutrophils and the formation of NETs. Subsequently, NETs support the invasion and metastasis of cancer cells. It has been found that CXCL5 production was mediated by DDR1-induced NF-κB signaling. Taken together, NETs take part in molecular circuits of a proinflammatory tumor microenvironment that ultimately may lead to PDAC invasion and metastasis [61].

**Figure 1:**
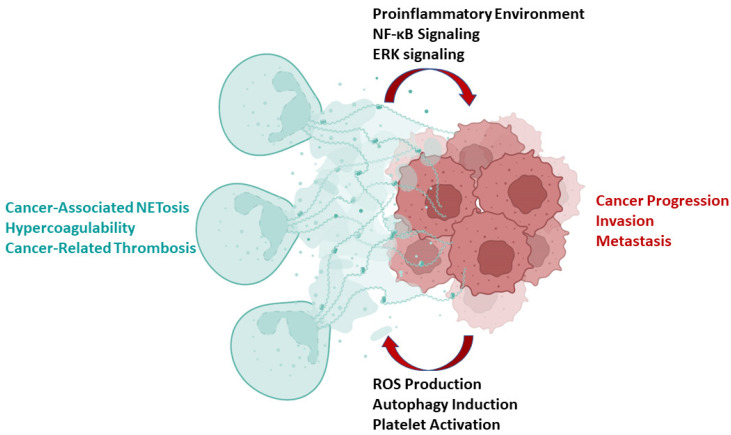
The vicious cycle between NET formation and cancer progression in PDAC. Activated neutrophils (green cells on the left) form NETs, as a result of NETosis. Cancer cells (red cells on the right) grow to form a tumor, partly by interacting with their microenvironment. Each arrow indicates a mechanism that results in NET formation or cancer progression, respectively. The end results of these mechanisms are also presented with color coding corresponding to each procedure (green for NETosis and red for tumor growth).

Taken together, the available data from human PDAC and several respective mouse models support the participation of NETosis and NETs in all stages of PDAC development, from the phenomenon of EMT to tumor formation, migration and metastasis. NET production is also associated with the clinical markers of poor prognosis and possible targets for future therapies of PDAC. An overview of the roles of NETs in PDAC progression is presented in Figure 1.

## 4. NETs and PDAC-Associated Hypercoagulability and Thrombosis

Cancer-associated thrombosis is among the most recognized complications during tumorigenesis [62,63]. Several studies reveal that cancer patients have at least a four- to seven-fold increased risk for the occurrence of venous thromboembolism (VTE) in comparison to the general population [64]. Importantly, the risk for VTE may significantly vary in different cancer types. For example, breast cancer patients have a low rate of VTE, while patients with PDAC have a high rate of VTE [65] and thus are at a higher risk of developing VTE [66]. Hypercoagulability may be induced following the formation of tumor-associated NETs [13]. This is also true in PDAC, since NETs that are formed following autophagy induction may promote hypercoagulability both in mouse models and in human PDAC [67].

Pancreatic cancer is among the neoplasias with the most metastatic potential [68]. NET production by activated polymorphonuclear neutrophils has been associated with platelet activation and reactive oxygen species (ROS) production in the tumor microenvironment in various cancer types, including glioma [69], lung cancer [70], breast cancer [71] and PDAC [72]. A study by Abdol Razak et al. has shown that PDAC can induce a rapid release of NETs in a platelet-dependent and ROS-independent manner. Significantly, as a consequence to PDAC development, the end result of NET release was venous thrombus formation [73].

In a clinical study of 83 patients with pancreatic cancer and 30 healthy individuals, NET formation was associated with procoagulant activity. It was found that in higher-stage disease, inflammatory factors are linked to NET formation and endothelial cell activation which induce a prothrombotic state on blood cells. Blood cells, microparticles and NETs have been found positive for phosphatidylserine (PS) in a stage-dependent manner and related to increased procoagulant activity in patients with PDAC [74]. NET release converts an endothelial cell to a procoagulant phenotype, contributing to the elevation of factor Xa (FXa), thrombin and fibrin formation levels and rapid coagulation in relation to healthy control samples. Notably, isolated NETs stimulate morphological changes in endothelium, such as retraction from cell–cell junctions and a biochemical alteration, by inducing DNase I and protein C activity. The authors suggested targeting either PS or NET production in PDAC to prevent thrombosis and cancer progression [74].

The role of neutrophils and NETs to enhance venous thrombosis has been validated in ex vivo xenografts of human PDAC tumors in mice [75]. In the aforementioned study, PDAC-bearing mice were positive for NETs markers such as citrullinated histone H3 (H3cit), the neutrophil marker Ly6G. Thrombosis was inhibited following neutrophil depletion or DNAse I administration. In another study, Maraucher et al. has shown that H3cit histone modification, an established marker of NET formation, is associated with venous thromboembolism only in patients with specific types of cancer, such as PDAC and lung cancer, but not in other types, such as breast carcinomas [76]. The production of NETs has a predictive value in PDAC, since post-operative recurrence in pancreatic neuroendocrine tumors is associated with NET formation and the presence of tumor-infiltrating neutrophils [77].

## 5. Therapeutic Potential

Given the dual role of NETs in both PDAC pathogenesis and VTE, neutrophil activation and NET formation hold promise for therapeutic interference.

To this end, Kajioka et al. have shown that thrombomodulin seems like a potential candidate that targets NETs and inhibits metastasis in pancreatic cancer [78]. The authors established a connection between high-mobility group box 1 (HMGB1), a protein that is present in NETs, and the induction of metastasis. HMGB1 is an important regulator of chromatin structure that possesses a dual role during pancreatic cancer development [79]. Intracellular HMGB1 may act as a tumor suppressor in the initial stages of PDAC, while extracellular HMGB1 can promote inflammation and metastasis [79]. The suggested mechanism by thrombomodulin includes the targeting and degradation of the HMG1 inhibition of NET formation and the suppression of metastasis as an end result [78].

Another pharmaceutical candidate is chloroquine. Chloroquine has been found to inhibit NET formation and the associated hypercoagulability in an orthotopic PDAC mouse model and samples from PDAC patients [80]. The presence of NETs in whole blood stimulates both the activation and aggregation of platelets, a result that has been verified using PAD4- and RAGE-knockout mouse models, which are deficient for NET formation. Chloroquine, which is a potent inhibitor of NET formation, significantly reduces platelet aggregation, the amounts of circulating tissue factor and hypercoagulability. This finding is supported by the fact that in acute pancreatitis the induction of NETs contributes to disease severity, while chloroquine administration inhibits both NETs and the associated phenotypes [81].

Apart from thrombomodulin and chloroquine, there are several more effective inhibitors of NETosis and NET formation. Such inhibitors fall into different categories, based on the demonstrated mechanism of action [82]. Agents such as acetylsalicylic acid target cyclooxygenase enzyme (COX) and consequently thromboxane A2. As a result, acetylsalicylic acid inhibits NETosis by disrupting platelet activation [83]. Anticoagulant agents contain several potent inhibitors of NETosis, such as inhibitors of protein C, including thrombomodulin [84] and activated protein C, or antithrombin, including heparin [85]. Since ROS production is a significant factor that contributes to NET formation, NETosis is also inhibited by antioxidant agents, such as N-acetylcysteine [86] and diphenyleneiodonium chloride [81]. The verified inhibitors of NETosis also hold a potential to disrupt the vicious cycle between NETs and PDAC.

## 6. Conclusions and Future Perspectives

Cancer development is the epitome of the deregulation of fundamental regulatory mechanisms at a cell and systems level. NETosis is a physiological acute response to infection which must be controlled in degree and duration. Here, we summarized the interplay between the deregulation of NET formation and tumor progression on pancreatic cancer. Together, they form a vicious cycle (Figure 1) that leads to a feedback mechanism with devastating consequences for the cancer patient. On the one hand, NETs provide a tumor-enabling substrate that instigates the proliferation, invasion and metastasis of PDAC. On the other hand, cancer development stimulates NET formation, promoting cancer-related thrombosis. The available data suggest that targeting NETosis would disrupt this feedback mechanism and may enhance survival and quality of life in patients with PDAC.
